# Cold atmospheric pressure plasma: simple and efficient strategy for preparation of poly(2-oxazoline)-based coatings designed for biomedical applications

**DOI:** 10.1038/s41598-020-66423-w

**Published:** 2020-06-11

**Authors:** Petra Šrámková, Anna Zahoranová, Jakub Kelar, Zlata Kelar Tučeková, Monika Stupavská, Richard Krumpolec, Jana Jurmanová, Dušan Kováčik, Mirko Černák

**Affiliations:** 1 Department of Physical Electronics, Faculty of Science, Masaryk University, Kotlarska 2, 611 37 Brno, Czech Republic; 2 Dapartment for Biomaterials Research, Polymer Institute, Slovak Academy of Sciences, Dubravska cesta 9, 845 41 Bratislava, Slovakia

**Keywords:** Biosurfaces, Polymer chemistry

## Abstract

Poly(2-oxazolines) (POx) are an attractive material of choice for biocompatible and bioactive coatings in medical applications. To prepare POx coatings, the plasma polymerization represents a fast and facile approach that is surface-independent. However, unfavorable factors of this method such as using the low-pressure regimes and noble gases, or poor control over the resulting surface chemistry limit its utilization. Here, we propose to overcome these drawbacks by using well-defined POx-based copolymers prepared by living cationic polymerization as a starting material. Chemically inert polytetrafluoroethylene (PTFE) is selected as a substrate due to its beneficial features for medical applications. The deposited POx layer is additionally post-treated by non-equilibrium plasma generated at atmospheric pressure. For this purpose, diffuse coplanar surface barrier discharge (DCSBD) is used as a source of “cold” homogeneous plasma, as it is operating at atmospheric pressure even in ambient air. Prepared POx coatings possess hydrophilic nature with an achieved water contact angle of 60°, which is noticeably lower in comparison to the initial value of 106° for raw PTFE. Moreover, the increased fibroblasts adhesion in comparison to raw PTFE is achieved, and the physical and biological properties of the POx-modified surfaces remain stable for 30 days.

## Introduction

Surface modification of polymers is crucial in many applications, where material must fulfill specific requirements concerning the surface wettability^1^, stiffness^2^, topology^3^ as well as chemical reactivity^4^. Adjusting the individual surface properties is critical, especially in medicine. Implantable devices or scaffolds must exhibit not only long-term stability in contact with the biological environment but also provide specific interactions with tissues, possess required mechanical or antibacterial properties, or release drugs on demand. Some applications require the material to be non-adhesive and rigid; in other cases, the good cell adhesion is desirable^5^. Such control of the specific surface properties of medical implants or devices can be ensured by surface modification^6^.

The basic strategy for surface modification of low-cost polymers often used in medicine (polypropylene, polyethylene, polytetrafluoroethylene) includes chemical coating by a thin layer of a biocompatible polymer. Besides the polyethylene glycol (PEG) considered being a gold standard for many biomedical applications including surface modification, poly(2-oxazolines) (POx) are gradually more discussed as an available alternative^7,8^. POx are accessible via cationic ring-opening polymerization (CROP), which is well controllable process providing the defined polymers with desired architecture, functional moieties, and adjustable properties. Their relevance for employing as biomedical coatings is supported by extensive biological studies of biocompatibility^9,10^, immunotoxicity^11,12^, and control of protein and cell adhesion^13^ with positive results.

Up to now, many different modification techniques have been applied to form a POx-coated surface, such as ‘grafting from’, ‘grafting to’ methods as well as effective photoinitiated procedures. ‘Grafting from’ process takes place by CROP polymerization initialized from the surface bearing suitable initiating moieties. This process provides usually brush-like POx layer, where linear or bottle-brush POx chains grow from the surface by chain mechanism^14,15^. On the contrary, ‘grafting to’ approach employs direct end-capping of living POx tails by an appropriate functionalized surface containing terminating groups, e.g., amine^13^. Another efficient approach for immobilization of POx layer represents photoinitiation process. Upon UV irradiation, benzophenone moieties attached to surface create diradicals able to react with pre-synthesized POx chains via C-H insertion^16^. Similarly, highly reactive singlet nitrene forming from the photolabile aryl azide moieties placed either on appropriate surface or in the POx backbone is able to bind to almost all hydrocarbon moieties^17,18^. In addition to mentioned brush-like POx layers, recently the cross-linked coatings with anti-biofouling properties were synthesized by photoinitiated radical crosslinking of linear POx polymers composed of acrylate groups^19,20^. However, these methods represent time-consuming synthetic procedures requiring the surface pre-treatment, functionalization, and purification after each consecutive step. In contrast to these intricate techniques, a recently published plasma polymerization introduced a more facile, straightforward, and surface-independent way for the preparation of POx-based coatings. Bhatt *et al*.^21^ and the group of Vasilev^22,23^ independently published successful plasma-induced polymerization of 2-oxazoline monomers under the low-pressure conditions. The adjustment of plasma treatment parameters allowed to control the resulting surface properties and the prepared POx coatings were non-cytotoxic towards human dermal fibroblasts and biofilm adhesion on these surfaces significantly decreased from >99% to 9.2%, which confirmed their anti-biofouling properties^24^. Moreover, such plasma-deposited POx coatings possessing anti-biofouling characteristics demonstrated possible applicability in medical diagnostics as well as for nanotopography surface fabrication^25–27^. Despite all attractive features of plasma-induced polymerization of 2-oxazolines, mentioned studies used plasma process operating at low pressure, where it is necessary to work in a closed chamber in the atmosphere of noble gas with toxic monomers potentially causing contamination of samples. Moreover, the excessive fragmentation occurring during the plasma polymerization causes low control over the process representing by a complex mechanism, which suggests the formation of a variety of chemical fragments and functional groups in the POx coating^28^.

In this work, we propose to overcome these disadvantages using well-defined POx-based copolymers prepared by living cationic polymerization as a starting material for the preparation of coatings, which are further post-treated by non-equilibrium “cold” plasma generated at atmospheric pressure to ensure the covalent bonding and stabilization of POx layer. As a substrate is selected polytetrafluoroethylene (PTFE) due to its relevance for application in medicine^29^ and as a source of non-thermal homogeneous plasma is used diffuse coplanar surface barrier discharge (DCSBD) operating at atmospheric pressure in the ambient air, which is suitable for the rapid treatment of temperature-sensitive materials like polymers^30^. Herein, we examine for the first time the influence of plasma post-treatment on defined POx-based coatings with respect to their wettability, chemical composition, stability and biological activity.

First, the DCSBD plasma pre-treatment process of PTFE is extensively investigated, in order to achieve optimal conditions for further POx layer deposition. Subsequently, we aim to compare the parameters of the plasma post-treatment process and the effect of two different working gases (air vs. argon), in order to find the most efficient conditions for preparation of covalently attached POx layer to PTFE surface. Next, we study the stability of prepared layers after washing and during 28 days of aging. In all these tests, the surfaces were thoroughly characterized by FTIR, XPS, WCA and SEM. Finally, the selected stable POx layers were tested *in vitro* by fibroblasts adhesion tests. The presented new method of PTFE surface modification combines the advantages of DCSBD plasma treatment, which operates at atmospheric pressure in ambient air, it is fast, and environmentally friendly, with the benefits of CROP polymerization of POx, which yields well-defined and biocompatible polymers, to achieve surface properties suitable for biomedical applications.

## Results and discussion

In this study, poly(2-oxazoline) (POx)-based coatings are deposited and post-treated by means of non-thermal homogeneous plasma generated by DCSBD^31,32^. DCSBD plasma is generated in a very thin layer (∼0.3 mm)^32^ and low temperature; thus, it is suitable for the rapid treatment of temperature-sensitive materials like polymers as well as biological material^33^. Polytetrafluoroethylene (PTFE) is selected as a substrate due to its relevance for medical applications and low overlapping character with characteristic POx bands in FTIR and XPS spectra. The resulting composition of statistical copolymer poly(2-methyl-2-oxazoline)-*stat-*(2-(3-butenyl)-2-oxazoline) (PMEOx) determined from ^1^H NMR spectrum (Fig. S2) confirmed the presence of 4% of EnOx in the copolymer structure, which almost corresponds with 5% in the feed. We have selected such statistical copolymer composed of MeOx and EnOx, since similar POx-based copolymers have been previously used e.g., for the preparation of UV-crosslinked hydrogels by our group^34^ as well as by other researchers^35^. Not only the initial copolymers, but even resulting hydrogels exhibited good biocompatibility^34^ and low fibroblast adhesion improved by modification with RGD peptide sequences^36^.

**Figure 1:**
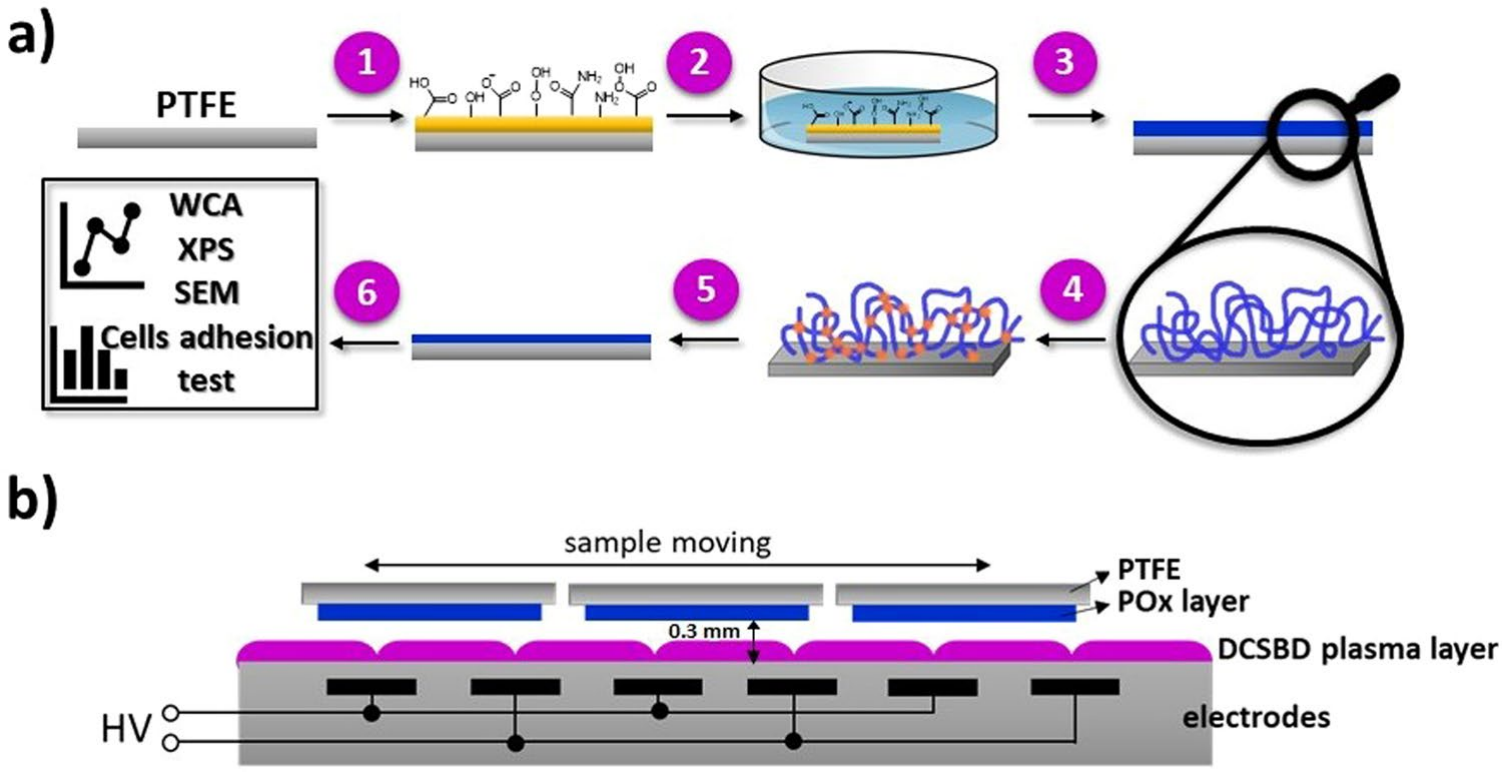
Schematic representation of experimental procedure and DCSBD plasma source. (**a**) Experimental procedure for preparation of PMEOx-based coating: (1) plasma activation of PTFE surface, (2) dip-coating of PMEOx layer, (3) drying, (4) plasma post-treatment of PMEOx layer (air and argon used as working gases), (5) washing in distilled water, (6) characterization; (**b**) Schematic cross-section of DCSBD plasma source with an illustration of sample moving perpendicularly to the axes of the electrodes.

The experimental procedure for preparation of PMEOx-based coating is depicted in Fig. 1a. PTFE surface is pre-treated by DCSBD plasma (1) to activate the surface (2) with the aim of covalently attach PMEOx layer through created radicals and various reactive groups on the surface. After drying (3), the PMEOx films are subsequently exposed to DCSBD plasma generated in air and argon (4). Since the stability of the prepared PMEOx layer is considered to be a critical parameter; the layer after plasma treatment is thus washed to remove all unattached material (5) and subsequently characterized (6). The main idea of this study is to find the optimal conditions of the plasma (pre-)treatment process, i.e., type of working gas and plasma exposure time for the preparation of stable covalently attached PMEOx layer on PTFE surface for biomedical applications.

### Plasma activation of PTFE surface

**Figure 2:**
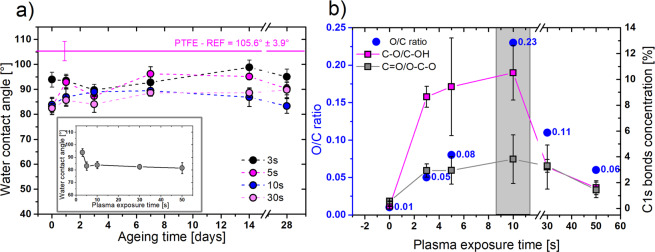
Optimization of PTFE plasma pre-treatment in air by WCA and XPS measurements. (**a**) Ageing behavior of air plasma-treated PTFE measured by WCA during the 28 days of storage. The inserted graph represents the WCA value development in dependence on the plasma exposure time. Mean values ± standard deviation from 12-16 droplets are plotted in the graph; (**b**) O/C ratio and concentration of O-based functional groups at the PTFE surface in dependence on the plasma exposure time, measured by XPS. Mean values ± standard deviation from 3 spots on the sample are plotted.

Prior to PMEOx layer deposition, it is necessary to activate the PTFE surface to create reactive groups enabling the attachment of PMEOx layer. Due to the high electronegativity of the fluorine atoms, the PTFE surface possesses extremely water repellent character. In our study, the water contact angle (WCA) of the pristine PTFE was 105.6° ± 3.9°, which confirms its highly hydrophobic nature. Plasma as a mixture of a large amount of different reactive species is capable of the breakdown of strong bonds in the PTFE structure and promotes the functionalization of the surface. As a result of the incorporation of polar functional groups, the surface gains less hydrophobic character which is related to the WCA decrease after the plasma treatment. Already after 3 s of the treatment we achieved the decreasing of WCA from initial 105.6° ± 3.9° to 93.9° ± 3° (Fig. 2a – inserted graph). Representative water droplets took at the surface of raw PTFE and plasma treated PTFE are depicted in Fig. S3. With longer plasma exposure time, WCA values slightly decreased. However, after 5 s of treatment, we observed an apparent saturation point. PTFE treated longer than 5 s (10–50 s) induced only negligible reduction of WCA to the lowest value of 81.5° after 50 s of treatment as it is displayed in Fig. 2a. Up to now, the treatment of PTFE by DCSBD plasma source was reported in few articles^37,38^, but the information about WCA changes was mentioned only by Károly *et al*.^39^ Here, the authors achieved a negligible decrease of WCA from initial 108° to 101° after 30 s of plasma treatment; however, their plasma setup parameters were slightly different. They used the longer distance between the sample and plasma source (0.5 mm) and lower input power (320 W) and these conditions were thus less efficient compared to our case. Considerable increase of PTFE wettability was observed in a study of Pavliňák *et al*.^40^ employing the surface dielectric barrier discharge generated in the air at the boundary line with the water solution. They achieved WCA value slightly above 60°. For evaluation of the stability of plasma-induced changes, we further monitored the development of wettability in time. Figure 2a displays the ageing process of treated PTFE during the first month. After two weeks, the WCA of all samples negligible increased; however, surprisingly after one month, WCA dropped down again which can be attributed to additional oxidation of the surface after the longer storage time. Despite the slight rise of WCA during the storage, observed values did not approach the initial WCA value of raw PTFE indicating the retention of polar functional groups on the surface.

Figure 2b shows O/C ratio and concentration of oxygen-based functional groups at the PTFE surface in dependence on the plasma treatment time measured by XPS. The maximal O/C ratio, which corresponds to the highest concentrations of oxygen-based functional groups, was achieved for intermediated treatment time of 10 s. The longer treatment time led to the decrease of the O/C ratio. Noteworthy, plasma-treated PTFE surface lacks nitrogen-based groups content, despite the use of air plasma. The absence of nitrogen in the similar experimental conditions was also described in an article published by Tóth *et al*.^41^ Considering the concentration of specific chemical bonds achieved from high-resolution C1s spectra (Table S1), concentration of CFx groups was decreasing from initial 96% while reached the minimal amount of 61% after 10 s of plasma treatment. This agrees with the highest obtained oxidation of PTFE after 10 s of treatment, which represents the boundary for the maximal degree of PTFE surface activation. For treatment times longer than 30 s, the concentration of CFx starts to increase again, which can be explained by possible etching of PTFE surface resulting in reducing of oxygen species on the plasma-activated surface as was previously reported elsewhere^38^. The results of XPS analysis, periodically repeated during the first 28 days, support the stability results achieved by WCA. The slight increase of concentration of polar functional groups as C-O/C-OH and C=O/O-C-O was observed, which corresponds to additional oxidation and possible contamination of the surface. In the case of sample treated for 10 s, the surface reached the highest concentration of polar functional groups after 28 days, which agree with the decreasing of WCA values (Table S2). Due to the highest concentration of oxygen-based functional groups revealed by WCA and XPS, we chose the 10 s as an optimal pre-treatment time of PTFE surface.

### Optimizing of plasma post-treatment of PMEOx layer

**Figure 3:**
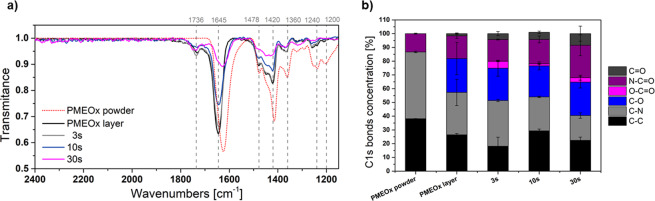
Optimization of plasma exposure time used for post-treatment of PMEOx coatings. (**a**) FTIR spectra of PMEOx powder (red dot line), PMEOx layer (black line) and treated PMEOx layer in different air plasma exposure times – 3 s (grey line), 10 s (blue line), 30 s (magenta line), without washing; (**b**) Relative concentrations of particular chemical bonds from high-resolution C1s spectra of air-plasma treated PMEOx layer as a function of plasma exposure time measured by XPS.

The pre-treated PTFE surface was coated with PMEOx layer by a dip-coating method and additionally treated by DCSBD plasma. First, we optimized the air plasma exposure time to achieve covalently attached and stable POx layer. We consider the influence of two competitive events during plasma treatment, etching and crosslinking. Concerning the high efficiency and volume power density of DCSBD plasma, usually a few seconds are enough to achieve desired chemical changes on the surface and to avoid the unfavorable etching. FTIR spectra in Fig. S4 and Fig. 3a compare PMEOx before and after air plasma treatment with raw PMEOx powder used for coatings. Fig. S4 in Supporting Information depicts the full range of FTIR spectra, but for a more detailed view, Fig. 3a represents the fingerprint range 2000–1100 cm^−1^. The spectra are recorded before washing. The spectrum of raw PMEOx (red dot line) contains a typical broad peak for the amide group at 1624 cm^−1^. Spectral range 1050-1350 cm^−1^ representing CH deformation region consists of three absorption bands^42^. Peak at 1478 cm^−1^ is assigned to CH_2_ deformations from the polymer backbone, broad shoulder at 1450 cm^−1^ corresponds to asymmetric CH_3_ deformations and symmetric CH_3_ deformations appears at 1360 cm^−1^. Broad bands with medium intensity around 1240 and 1200 cm^−1^ can be assigned to C-N stretching vibrations^43^. Already after the PMEOx deposition on pre-treated PTFE by DCSBD plasma (black line), characteristic signal for amide shifted to 1645 cm^−1^ and the new peak at 1736 cm^−1^ corresponding to C=O group in carboxylic acid appeared. We assume that plasma activated PTFE substrate induces chemical changes in PMEOx structure resulting in covalent binding of PMEOx layer already after deposition as well as appearing of new functional groups even without additional plasma post-treatment, which explains the presence of O-C=O group signal in FTIR spectra. This absorption band represents the sole difference in the spectra of PMEOx powder and deposited PMEOX layers after PTFE pre-treating. Beside this, we noticed the decreasing intensity of other typical PMEOx bands with increasing of the plasma treatment time, which demonstrates considerable etching of the PMEOx layer and thus the removing of the upper layer of PMEOx film. In contrast to negligible changes in FTIR spectra reported here (see Fig. S4), POx coatings prepared by plasma-induced polymerization exhibited the presence of additional functional groups, such as isocyanates (2170 cm^−1^), nitriles (2250 cm^−1^) as well as broadening of amide bands^22^.

FTIR results are supported by high-resolution C1s spectra measured by XPS (Fig. 3b), where the concentration of C-C and C-N bonds declines at the expense of forming new C-O, C=O and O-C=O bonds. Appearing of signal for carboxylic acid group in C1s spectra agrees with FTIR results, except the case of PMEOx layer without plasma post-treatment. Concentration of N-C=O bonds rose with plasma exposure time, while intensity for amide group signal in FTIR decreased. In comparison to FTIR analyzing the deeper surface profile, XPS monitors a thin upper layer of the surface (3–10 nm); hence they cannot be directly compared. Here, it should be noted that the thickness of the deposited PMEOx layer was only estimated from indirect measurements, due to the restricted access to other direct techniques, such as ellipsometry. According to SEM images depicted in Fig. S5, we assessed the average thickness of the deposited PMEOx layer to 1–3 μm. Since our primary goal was to attach and stabilize the PMEOx layer, we decided to use 3 s as appropriate plasma exposure time to avoid undesired etching.

**Figure 4:**
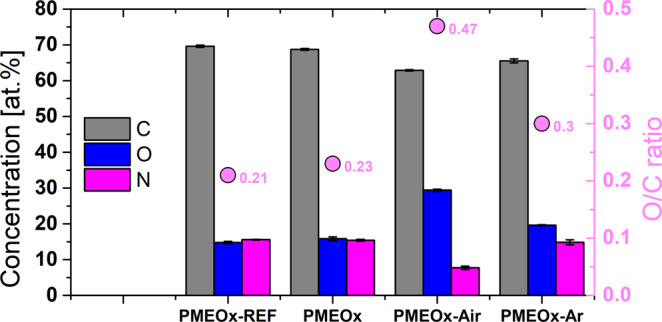
Atomic concentration of PMEOx coatings achieved in different plasma treated conditions measured by XPS. Comparison of atomic concentration (%) of C, O, N in PMEOx coating before (sample PMEOx) and after plasma treatment (samples PMEOx-Air, PMEOx-Ar) with PMEOx-REF.

PMEOx coating immediately after the deposition as well as after the plasma post-treatment in air and argon were highly hydrophilic, hence the WCA values could not be obtained. Similar behavior of spin-coated MeOx-based thin film was discussed in a recently published article^44^. The WCA value increased with the decreasing polarity of linear poly(2-oxazolines) deposited on the substrate. In the case of the film based on MeOx, it achieved 0° due to its most polar character which agrees to our results. As the chemical structure of raw PMEOx is represented by molecular formula C_4_H_5_NO, the relative theoretical percentage of elements C, N, O is 66%, 17%, 17%, respectively. The Fig. 4 summarizes the atomic percentage of individual elements presented in PMEOx coatings before and after plasma treatment. The atomic composition of PMEOx layer (PMEOx) immediately after deposition as well as the reference PMEOx layer (PMEOx-ref) are similar and correspond well with theoretical values. We monitored slight increase of oxygen concentration after plasma generated in argon (20%), but the more pronounced increase was observed in the case of air plasma (29%). Additionally, we monitored the decrease of nitrogen concentration (N/C ratio decreased from 0.22 to 0.12) after air-plasma treatment, which is a consequence of the high tendency of bonds cleavage in the plasma generated in air.

Table 1 shows the relative percentage of individual chemical bonds obtained from high-resolution C1s spectra. Even the simple deposition of PMEOx layer on activated PTFE without the plasma post-treatment induced the chemical changes, specifically the concentration of C-C/C-H bonds decreased while the concentration of amines, ester, carbonyl and amide groups increased. Noticeable cleavage of C-C/C-H bonds in the plasma generated in air confirmed its highly destructive character in comparison to argon plasma. More pronounced oxidation in air plasma is support by the presence of 5% of carboxyl groups, which appeared only under these plasma treatment conditions.

**Table 1:** The relative percentage of chemical bonds observed from high-resolution C1s spectra of PMEOx samples before and after washing.

Sample	Before washing				After washing			
	C1s bonds concentration [%]				C1s bonds concentration [%]			
	C-C/C-H	C-O/C-N	C=O/N-C=O	COO	C-C/C-H	C-O/C-N	C=O/N-C=O	COO
PMEOx-ref	38	49	13	—				
PMEOx	26	56	18	—	13	53	19	15
PMEOx-air	18	57	20	5	13	58	17	12
PMEOx-Ar	23	54	23	—	30	54	6	10

PMEOx reference layer is compared to samples without plasma post-treatment (PMEOx), treated by air-plasma (PMEOx-air) and argon-plasma (PMEOx-Ar). The values of concentrations of chemical bonds after washing are recalculated with exclusion of CF_x_ bonds.

### Stability of POx layer

**Figure 5:**
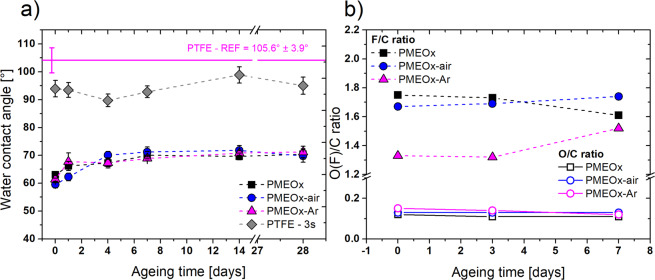
WCA and chemical composition development during storage. (**a**) WCA values of washed PMEOx coatings and their ageing behavior during the 28 days of storage compare to raw PTFE treated by plasma at 3 s; (**b**) Changes in O/C and F/C ratios of PMEOx coatings during the 7 days of storage.

Firstly, the stability of the PMEOx layer after washing the samples in water was investigated by WCA measurements. Surprisingly, a thin layer was formed already after the deposition of PMEOx on activated PTFE surface (sample PMEOx) without plasma post-treatment and for all samples (PMEOx, PMEOx-air, PMEOx-Ar) the observed wettability was very similar. Achieved WCA values were around 60° (Fig. 5a), which is considerably lower in comparison to plasma-treated raw PTFE (see Fig. S3). As a reference value, we selected PTFE after 3 s of plasma treatment, since we used this exposure time for the post-treatment of PMEOx layer. For raw PTFE WCA of 93.9° (Fig. 2a) was observed after plasma treatment in ambient air and the value 90.5° was achieved in argon (data not shown). Even the additional rinsing in water did not have an effect on WCA of plasma-treated PTFE; the values were still higher than 90° (3 s of plasma treatment – 96.3° ± 3°, 30 s of plasma treatment – 90.5° ± 5.4°). In the previous articles dealing with plasma polymerization of POx on the particular surface (glass coverslips, silicon wafers), the WCA reached the values in the range of 52-62° after rinsing with water and were dependent on the input power used for plasma deposition^22,23^. Similar results were published by Van Guyse *et al*^44^. of the authors used different 2-oxazoline monomers obtaining a narrow range of WCA values between 50-60° which means that the polarity of initial monomer does not influence the wettability of resulting POx coating. Since we used MeOx-based polymer for preparation of all POx-based coating, the observed comparable WCA values for the sample without plasma treatment (PMEOx - 63°) and after plasma post-treatment (PMEOx-air – 59.5°, PMEOx-Ar – 61.4°) corroborates previously published data.

In Fig. 5a is depicted the development of WCA during the 28 days of storage and proves the high stability of PMEOx layers. In all cases, WCA slightly increased to 70°-71°; however, these values still represent the hydrophilic character of the POx-modified surface. In a recent study on the stability of plasma polymerized POx coatings, a competitive behavior of two opposite processes was discussed, namely hydrophobic recovery and oxidation during the storage of samples in defined conditions^45^. They monitored a negligible increased of WCA from 64° to 68° for the samples stored at ambient conditions without the access of light due to the post-deposition stabilization involving additional crosslinking as well as charge species recombination in the coating. In our case decreasing in hydrophilicity was caused by more pronounced hydrophobic recovery represented by rotation of polar functional groups towards the bulk of the PMEOx layer. Accordingly, the hydrophobic recovery overrode the effect of oxidation which was proved also by results of chemical composition measured by XPS, which will be discussed later.

Washed PMEOx coatings were characterized also by XPS immediately after washing as well as in the ageing period of one week, to study the chemical changes of their uppermost surface layer. It is important to say that after washing, the signal for F atom still appeared in survey spectra as a result of detected photoelectrons from PTFE substrate. This observation led us to the assumption that the thickness of the resulting POx layer is less than 10 nm corresponding to the maximal depth of XPS measurements (Table S3). However, it should be noted that the direct measurement of the thickness of PMEOx layer after washing was not performed. High-resolution C1s spectra are depicted in Fig. S6. O/C ratios for all three samples are similar (0.12, 0.13, 0.15 for PMEOx, PMEOx-air, PMEOx-Ar, respectively) and they are higher than the value of plasma-treated PTFE at 3 s (0.05). The F/C ratio decreased from the initial 2.1 (raw PTFE) to 1.8, 1.7 and 1.3 for samples PMEOx, PMEOx-air, and PMEOx-Ar, respectively. These differences in O/C and F/C ratios confirm the retention of a thin PMEOx layer on the surface after washing. Additional evidence of the PMEOx layer is the presence of nitrogen on the surface which originates preferentially from the PMEOx. While after the plasma pre-treatment, no nitrogen was determined in the XPS survey spectra, the PTFE modified by PMEOx coating includes also N-based functional groups. Figure 5b representing the elemental F/C and O/C ratio reflects the high chemical stability of the PMEOx coatings regarding the unchangeable O/C values during the 7 days of storage which supports also the negligible changes in WCA. In contrast, the F/C ratio slightly oscillates, but it is within the errors of measurement.

**Figure 6:**
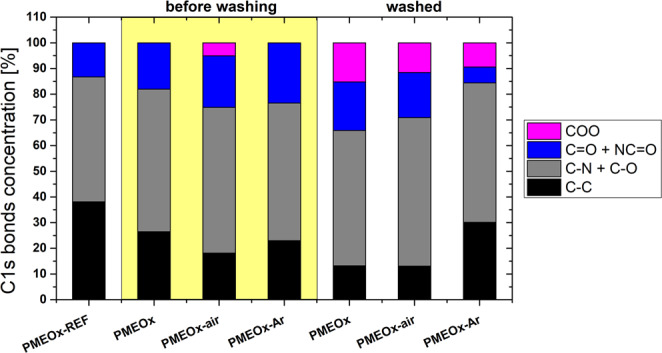
Relative concentrations of particular chemical bonds from high-resolution C1s spectra. Comparison of chemical composition of non-treated (PMEOx) and plasma treated (PMEOx-air, PMEOx-Ar) layer before and after washing to PMEOx-REF.

To compare the bonds concentration solely from the PMEOx layer after washing we re-calculated the percentage of bonds with the exclusion of all the CF_x_ bonds originated from the PTFE substrate. The resulting composition of the PMEOx layer after washing is depicted in Fig. 6 and Table 1, where is the percentage of appropriate chemical bonds compared to the case before washing. We observed the decrease of C-C/C-H bonds concentration for PMEOx layer prepared without plasma post-treatment and air-plasma treated layer (PMEOx-air) compared to the reference and to the samples before washing. The destructive character of air plasma may induce the degradation of PMEOx chains by cleavage of C-C bonds or hydrogen separation and additional binding of active functionalities present in dense plasma into the cleaved positions can occur. This results in higher percentage of carboxylic (COO), carbonyl (C=O), amide (NC=O), or C-O/C-N groups observed at surfaces of both samples. On the contrary, although the argon plasma consisting of radicals formed due to Ar^+^ or electron impacts can also cause the disruption of C-C/C-H bonds, in this case, the mutual interactions between the formed radicals predominantly occur. This results in cross-linking which is connected to higher concentration of C-C/C-H bonds for PMEOx layer post-treated in argon. In comparison to samples measured immediately after PMEOx deposition and/or additional plasma post-treatment, the washed samples consist of a high concentration of COO groups (9-15%) formed probably by additional hydrolysis in water.

### Surface morphology

**Figure 7:**
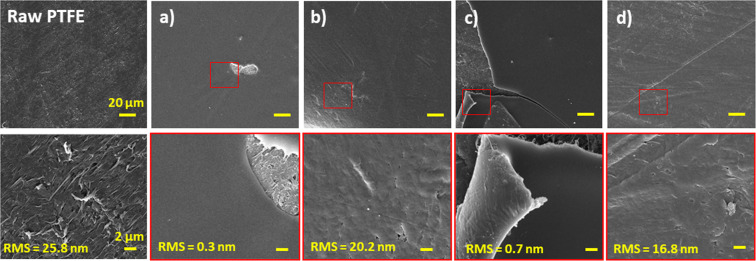
SEM images with inserted yellow values of roughness measured by AFM. Morphology and roughness of raw PTFE is compared to (**a**) PMEOx layer deposited after PTFE pre-treatment, (**b**) PMEOx layer deposited on pre-treated PTFE after washing, (**c**) air-plasma treated PMEOx layer, (**d**) air-plasma treated PMEOx layer after washing.

The roughness of the surfaces was studied by atomic force microscopy (AFM) and the morphology was determined by scanning electron microscopy (SEM). The surface of raw PTFE is visibly rough with value of roughness around 25.8 nm (Fig. 7). Deposition of PMEOx layer (Fig. 7a) flattened the surface and thus induced decreasing of this value to 0.3 nm. Additional air-plasma treatment causes minimal (in the mean of method sensitivity) increase of roughness (0.7 nm), accordingly, no visible morphological changes are observed (Fig. 7c). SEM micrographs of deposited PMEOx layer (Fig. 7a,c) depict places with visible damage purposely. Due to the smooth character of the samples, it was difficult to focus the electron beam. Despite this, the PMEOx layer appears to be very smooth and homogeneous before as well as after plasma treatment. Washed samples depicted in Fig. 7b,d confirm the presence of the PMEOx layer on the PTFE surface, since the morphology visibly changed and gained a smoother nature compare to PTFE. However, in both cases (washed samples without and with air-plasma post-treatment), the morphology of the PMEOx layer is very similar, which proves the washing out of upper unattached material. It means that the plasma post-treatment step did not ensure stable attachment of the whole PMEOx layer.

### Fibroblast adhesion

**Figure 8:**
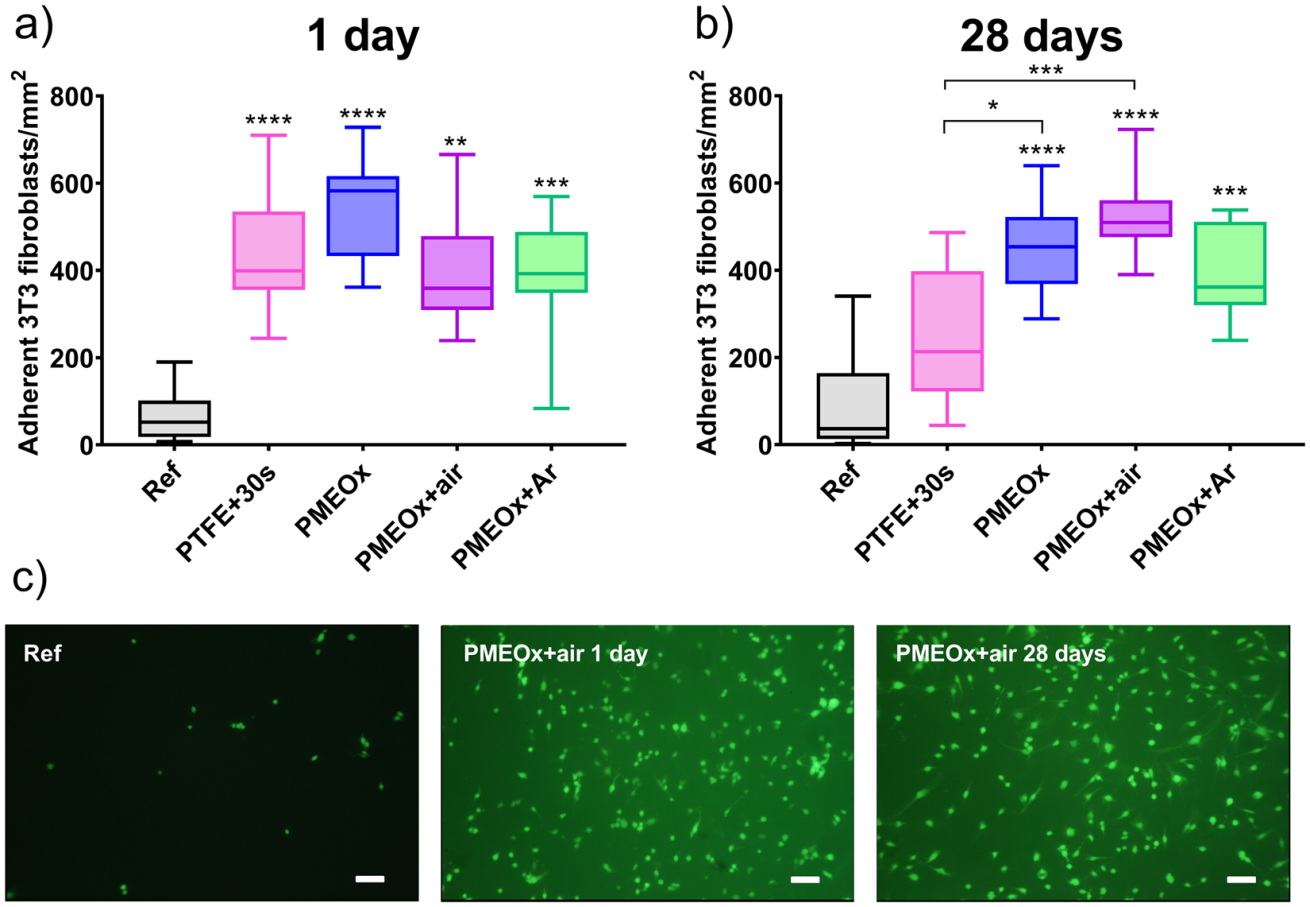
Study of the adhesion of 3T3 fibroblasts on PTFE and on POx surfaces after 24 h incubation. (**a**) Density of adherent cells on selected samples (*Ref* - raw PTFE, *PTFE* + *30* *s* - PTFE after 30 s of air plasma treatment, *PMEOx* – without plasma post-treatment*, PMEOx* + *air –* plasma post-treatment in air*, PMEOx* + *Ar* – plasma post-treatment in argon) 1 day after preparation. The box represents the median values and interquartile ranges of the number of adherent cells per mm^2^, the whiskers represent minimal and maximal values. **p < 0.01, ***p < 0.001, ****p < 0.0001, compared to ref (**b**) Density of adherent cells on selected samples after 30 days of aging. *p < 0.05, ***p < 0.001, ****p < 0.0001, compared to ref or *PTFE* + *30* *s*, (**c**) Representative fluorescent images of adherent cells stained by green FDA. Scale bar = 50 μm.

Further, we focused on the study of the adhesion of mammalian cells on the selected samples. As an appropriate model cell line to study biomaterial-cell interaction, we selected 3T3 mice fibroblast cell line. Fibroblasts are the cells of connective tissue, and they are also responsible for the scaring process. They play crucial role in the interaction of foreign material with the body, being the first cells to attach to the healing wound or implanted biomaterial^46^. Figure 8a shows the number of adherent fibroblast cells on studied surfaces one day after the preparation. The number of adherent cells on a control PTFE surface without the plasma treatment is quite low, with the median value of 69 cells·mm^−2^. Comparably low cell adhesion was also confirmed by other authors^47,48^. After the air plasma treatment of PTFE surface, the number of adherent cells significantly increased to 434 cells·mm^−2^ (median value). This observation corroborates the works of Reznickova *et al*.^49^ and Slepicka *et al*.^50^, where the authors achieved increased cell adhesion on plasma-treated PTFE surfaces. The interactions at the cell-surface interface are affected by number of factors, such as surface chemistry, wettability and/or roughness. Although the wettability after 10 s of air-plasma treatment increased, the achieved WCA value of 83.9° is still relatively high at the border between hydrophilic and hydrophobic nature of the surface. However, after 10 s of plasma treatment, we observed the most pronounced functionalization of PTFE surface by polar functional groups (Fig. 2b) which can promote the cell adhesion.

The significantly increased fibroblasts adhesion compared to the untreated PTFE surfaces was observed also in case of all PMEOx-modified surfaces, independently from the preparation method and used type of plasma. However, among different plasma-treated groups of samples, we did not observe significant differences. The number of adherent cells per mm^2^ ranged from 387 to 533 on these surfaces. This is rather surprising finding, since hydrophilic POx are generally considered to be excellent polymers for non-biofouling surfaces, comparable to poly(ethylene glycol)^51,52^. To name a few examples, poly(2-methyl-2-oxazoline)-grafted surfaces^53^, hydrophilic POx bottle-brush brushes^54^ and hydrogels^36^ were shown to diminish the cell adhesion. On the other hand, it should be mentioned that the plasma polymerized POx usually promote good cell adhesion^44,55^, however, their chemical structure differs significantly from POx prepared by living polymerization methods. Surprisingly, the ongoing discussion on these different adhesion properties is often limited to comparison of chemistry of the surface layer, i.e., hydrophilic vs. hydrophobic POx, presence of certain functional groups, the molar mass of the polymer chain. Another possible effect is the conformation of polymer chains on the surface. It was show that oligo(ethylene glycol) chains grafted on the Au or Ag substrates exhibit different conformation, what leads to differences in water molecule penetration and subsequently, differences in protein adhesion^56,57^. The similar reasons, i.e., lack of flexibility of POx polymer chains, may lead to the increased adhesion of cells also in our case, in comparison to non-biofouling POx surfaces reported in literature. However, the confirmation of this effect needs further examination.

As mentioned above, various chemical and physical properties of the surfaces affect the cell adhesion. The introduction of the polar functional groups, such as carboxyl, hydroxyl and amine on inert surface increase the hydrophilicity and promote the cell adhesion^55,58^. XPS measurements revealed the appearance of oxygen containing functional groups on POx surfaces. We thus assume that these groups contribute to increased cell adhesion found in our experiment. The wettability also plays an important role. The study of Lee *et al*.^59^ proved that fibroblasts adhere most on the surface with moderate wettability possessing WCA around 60° which corresponds to our PMEOx layers. Also, the morphology and the roughness of the surface affect the cell adhesion^60^. It was shown that the human fibroblasts attach and proliferate better on smooth surfaces (Ra~0.2 μm). PMEOx layers in our study have the roughness in order of tens nanometers (Fig. 7 – 20.2 nm, 16.8 nm) which can be considered as smooth surfaces. However, there is no difference in roughness for control PTFE samples and POx modified surfaces, we thus assume that the observed change in cell adhesion properties was caused by previously discussed parameters (wettability, chemical changes, polymer chain configuration), rather than by morphology of the surface.

Further, we studied the adhesion of fibroblast cells on the surfaces after 28 days of aging (Fig. 8b). The motivation for this study is the already discussed potential effect of ageing of plasma treated surface. In comparison to plasma treated PTFE surface one day after the preparation, after 28 days of aging, the number of adherent cells decreased to 251, approaching the value of controlled untreated sample. This leads us to the assumption that the plasma treatment effect is not permanent, but eventually leads to the recovery of original properties. On the other hand, POx surfaces after 28 days of aging in all cases exhibit similar increased adhesion of fibroblasts cells, which is significantly higher than the reference value. For better imagination of differences in adhesion, look at Fig. 8c representing fluorescent images of adherent cells stained by green FDA. These results are supported by the slight changes in WCA of PMEOx layer and almost no changes in chemical composition during the storage as was reported in the previous chapter. Moreover, these results prove the successful preparation of stable POx layer on PTFE surface, which maintains its cell-adhesive properties even after 28 days.

## Conclusion

In this work, we present a new strategy for the preparation of bioactive poly(2-oxazoline) based coatings at the surface of chemically inert PTFE by using effective “cold” plasma generated by DCSBD at atmospheric pressure. By means of this diffuse plasma characterized by high volume power density we succeeded in activation of raw PTFE surface already after 5 s of plasma treatment, associated with the decrease of WCA from 106° to 81.5°. Aiming to improve the control over the surface chemistry in comparison to plasma polymerization, we used well-defined POx based copolymer synthesized by conventional living cationic polymerization. We investigated the effect of additional post-treatment by plasma generated in ambient air and argon, which creates different active species. To avoid the unfavorable etching of PMEOx layer, the plasma exposure time intended for post-treatment was adjusted to 3 s. Further, we studied the stability of coatings after rinsing in water. Even though the unattached polymer chains of PMEOx coating were washed away, the remaining thin PMEOx layer preserved its properties and high stability. WCA of PMEOx layer achieved the lower value of 59.5° demonstrated the considerable enhancement in wettability in comparison to raw as well as plasma-treated PTFE substrate. Ageing effect during 28 days of storage was negligible since the WCA of PMEOx layers increased only to 70°, as well as the chemical composition was retained. While surfaces grafted by hydrophilic POx possess anti biofouling properties with minimal cell adhesion, our plasma driven prepared PMEOx layers exhibited increased fibroblasts adhesion and these cell-adhesive properties remained unchanged even after 28 days. It is in good accordance with recently published paper reviewing the POx coatings prepared by plasma polymerization^44^. Plasma post-treatment in a particular atmosphere influenced chemical composition of the layer, which was more pronounced in the case of argon plasma. However, it has only a minor effect on the resulting properties of PMEOx layer. We achieved very similar behavior related to wettability, ageing effect and cell adhesion properties, wherein the PMEOx layer maintained its stability in all cases. To conclude, we found out that POx coating prepared from well-defined polymers can be covalently attached to the inert PTFE substrate by means of DCSBD plasma, which effectively activated the surface under the optimized conditions. Moreover, our strategy for such modification can be even simplified by the exclusion of the plasma post-treatment step. The presented new method of PTFE surface modification combines the advantages of DCSBD plasma with the benefits of well-defined POx copolymers as a starting material for the preparation of hydrophilic, bioactive and stable coatings suitable for biomedical applications.

## Experimental section

### Material

Methyl 4-nitrobenzenesulfonate, diisopropylamine, allyl bromide and butyllithium in hexanes (2.5 M) were purchased from Sigma-Aldrich (Steinheim, Germany). 2-Methyl-2-oxazoline (MeOx) provided by TCI (TCI Europe N.V., Belgium) was dried over KOH for 48 h, distilled over CaH_2_ under the reduced pressure and stored under argon. Acetonitrile (J.T. Baker, Netherlands, HPLC grade) and dichloromethane (Lach-Ner, Czech Republic) were distilled over CaH_2_ and stored on molecular sieves under argon. Tetrahydrofuran (Centralchem, Slovakia) was distilled from sodium benzophenone ketyl prior to use. Isopropanol (Penta s.r.o., Czech Republic) and chloroform (Lach-Ner, Czech Republic) were used as received. For washing of samples before and after plasma treatment deionized water was used.

Polytetrafluoroethylene (PTFE) sheets with a thickness of 1 mm provided by Goodfellow Cambridge Ltd. were used as a substrate material for the deposition of the poly(2-oxazoline) layer. Before experiments, PTFE sheets were cut into different shapes appropriate for individual measurement techniques (squares with dimensions of 1 × 1 cm^2^, rectangles with dimensions of 1 × 2.5 cm^2^, circular samples with a diameter of 15 mm). Before all experiments, PTFE samples were ultrasonically cleaned in isopropanol and distilled water, in both solvents 2 times for 10 min and then dried in the nitrogen flow.

### Synthesis of 2-(3-butenyl)-2-oxazoline (EnOx)

2-(3-Butenyl)-2-oxazoline (EnOx) was prepared according to the procedure described in the work of Dargaville *et al*. (Scheme S1)^35^. In a pre-dried round-bottom flask, a lithium diisopropylamide (LDA) solution was freshly prepared as follows: dried tetrahydrofuran (200 mL) and dried diisopropylamine (24.8 mL, 0.177 mol, 1 equiv) was mixed and cooled to −78 °C under the argon atmosphere. Next, butyllithium in hexanes (67 mL, 0.168 mol, 0.95 equiv) was added dropwise into the cooled mixture. After stirring for 30 min, 2-methyl-2-oxazoline (15 ml, 0.177 mol, 1 equiv) was added into the reaction mixture and stirred for another 60 min. Further, allyl bromide (14 mL, 0.162 mol, 0.92 equiv) was added dropwise into the reaction mixture, stirred for 15 min, then the reaction mixture was left to equilibrate to the room temperature. After stirring overnight, 30 mL of methanol was added to quench the reaction. The volatiles were evaporated under reduced pressure, then 100 mL of dichloromethane was added. The mixture was washed 2 times with distilled water and 2 times with brine. After evaporating under reduced pressure, the product was obtained as a colorless liquid (17.84 g, 81%). Before the use in polymerization, the monomer was distilled over CaH_2_. For the ^1^H NMR spectrum of EnOx, see Fig. S1 in Supplementary Information.

^1^H NMR (400 MHz, CDCl_3_, δ, ppm): 5.73–5.82 (m, 1H, CH_2_=CH−), 4.91–5.02 (m, 2H, CH_2_=CH−), 4.14 (t, 2H; CH_2_–O), 3.74 (t, 2H; CH_2_–N), 2.3 (s, 4H; -CH_2_-CH_2_-).

### Synthesis of poly(2-methyl-2-oxazoline)-*stat*-(2-(3-butenyl)-2-oxazoline) (PMEOx)

A statistical copolymer poly(2-methyl-2-oxazoline)-*stat*-(2-(3-butenyl)-2-oxazoline) (PMEOx) was synthesized by the living cationic ring-opening copolymerization according to previously published procedure (Scheme-S2)^34^. In a pre-dried Schlenk flask under an argon atmosphere, distilled and dried acetonitrile (20 mL) as a solvent and methyl 4-nitrobenzenesulfonate (MeONs) as an initiator (0.217 g, 1 mmol), 2-methyl-2-oxazoline (8.09 g, 95 mmol), 2-(3-butenyl)-2-oxazoline (0.63 g, 5 mmol) was added. The reaction mixture was heated to 80 °C and polymerized for 24 h. The feeding ratio of monomers 2-methyl-2-oxazoline / 2-(3-butenyl)-2-oxazoline in the polymerization mixture was adjusted to 95/5 and degree of polymerization to 100. The reaction mixture was cooled on ice and terminated with KOH in methanol for 4 h. The copolymer was purified by precipitation into diethyl ether, followed by dialysis from water and freeze-drying. Statistical copolymer PMEOx was obtained as a white powder (yield 7.34 g, 84%). For the ^1^H NMR spectrum of PMEOx, see Fig. S2 in Supplementary Information.

^1^H NMR (400MHz, CDCl3, δ, ppm): 2.01–2.10 (bp, 3H, COCH_3_); 2.30−2.41 (bp, 4 H, COCH_2_CH_2_CH); 3.39 (bp, 4H, NCH_2_CH_2_); 4.92 − 5.01 (m, 2H, CH=CH_2_); 5.68–5.83 (m, 1H, CH=CH_2_).

### Plasma treatment

Activation of the PTFE surface, as well as post-treatment of deposited PMEOx layer, were performed by non-thermal plasma generated by diffuse coplanar surface barrier discharge (DCSBD) at atmospheric pressure. DCSBD is a unique type of coplanar dielectric barrier discharge further developed in our R&D Center^31,32^. It represents a planar electrode system consisting of 16 pairs of silver electrodes aligned in parallel and embedded in alumina ceramics 0.6 mm below its surface. The electrode system was powered by AC with high voltage up to 20 kV (peak-to-peak) at a frequency of about 15 kHz. Macroscopically uniform DCSBD plasma is generated in the form of thin plasma layer on the surface of flat dielectric ceramic in the form of numerous H-shaped microdischarges burning between the strip electrodes.

### PTFE activation

PTFE samples placed at 0.3 mm distance from the electrode were treated by DCSBD operating in ambient air at atmospheric pressure and it was supplied by sinusoidal voltage at input power 400 W. Samples were treated in dynamic mode (sample moving direction is depicted in Fig. 1b) in selected plasma exposure times (3, 5, 10, 30 and 50 s). Immediately after plasma treatment, PTFE samples were immersed in 5 wt.% PMEOx solution in chloroform for 1 min. Then, PTFE samples were removed from the solution by tweezers and dried at laboratory conditions overnight. The formed PMEOx layer was characterized and further treated by plasma.

### PMEOx layer treatment

The procedure was very similar as in the case of PTFE surface activation. PTFE samples with deposited PMEOx layer were treated by plasma in standard distance of 0.3 mm in a dynamic mode. For the treatment of samples in ambient air, the input power was set at 400 W (15 kHz), and plasma exposure time ranged between 3–30 s. The plasma treatment of PMEOx films in argon atmosphere was performed in a sealed reactor consisting of the treatment chamber with the DCSBD plasma source, as was previously described^61^. PMEOx-deposited PTFE samples attached to the holder were placed in the specific moving carrier, and the reactor chamber was closed. Reactor chamber with the inner volume about 2 L was carefully sealed, which allowed the filling of the inner space with argon flow a rate of 1.0 L·min^−1^. The reactor chamber was purged by argon for 5 minutes before the plasma treatment. The plasma was generated at an input power of 160 W (15 kHz) for exposure time of 3 s.

### Surface analysis

#### Nuclear magnetic resonance (NMR) spectroscopy

NMR spectroscopy was used for the characterization of structure of monomer and statistical copolymer. ^1^H NMR spectra were measured at room temperature in deuterated chloroform (CDCl_3_) on Varian 400-MR spectrometer (Varian, USA) using tetramethylsilane (TMS) as an internal standard.

#### Water contact angle measurements (WCA)

Water contact angle (WCA) measurements were carried out with a Surface Energy Evaluation System (See System) analyzer (Advex Instruments, Brno, Czech Republic) using 1 µL sessile droplets of deionized water. Resulting WCA values were calculated as the average of contact angles of 16–20 droplets, whereby the two highest and two lowest values were removed prior to evaluation.

#### Fourier transform infrared spectroscopy (FTIR)

Infrared spectra were measured by Bruker Vertex 80 V spectrometer in attenuated total reflection (ATR) mode using a diamond crystal as was previously described^62^. All measurements were made at a vacuum of 2.51 hPa to suppress undesirable effects caused by the presence of ambient humidity and CO_2_. Spectra were collected at a resolution of 4 cm^−1^ in range 4000–600 cm^−1^ using 100 scans for each sample and evaluated by OPUS software (version 6.5, Bruker) using ‘rubber-band’ baseline correction. The spectra of PMEOx layer and plasma treated PMEOx (3 s, 10 s, 30 s) were measured at Si wafer substrate to avoid the overlapping of signals for C-N (1200–1240 cm^−1^) with the strong signal for C-C in PTFE spectrum.

#### X-ray photoelectron spectroscopy (XPS)

Chemical changes at a particular surface (PTFE, PMEOx layer) immediately after the plasma treatment, as well as the ageing of the PMEOx layer in time, were monitored by XPS. The XPS measurements were done on the ESCALAB 250Xi (ThermoFisher Scientific). The system is equipped with 500 mm Rowland circle monochromator with a micro-focused Al Kα X-Ray source. An X-ray beam with 200 W power (650 microns spot size) was used. The survey spectra were acquired with pass energy of 50 eV and energy step of 1 eV. High-resolution scans were acquired with pass energy of 20 eV and energy step of 0.1 eV. In order to compensate the charges on the surface electron flood gun was used. Spectra were referenced to the hydrocarbon type C1s component set at a binding energy of 284.8 eV. The spectra calibration, processing, and fitting routines were done using Avantage software (Thermo Scientific)^63^.

#### Scanning electron microscopy (SEM)

Imaging of surface morphology was done using Scanning Electron Microscope MIRA3 (Tescan, Brno, Czech Republic) with maximal resolution 1 nm. The detector of secondary electrons and an accelerating voltage of 10 kV was used. To prevent any charging of the sample, samples were coated by 20 nm of Au/Pd composite layer^64^.

#### Atomic force microscopy (AFM)

Atomic force microscopy (AFM) measured by Ntegra prima (NT-MDT) microscope in semi-contact mode was carried out to measure the root mean square (RMS) surface roughness of the PTFE as well as the poly(2-oxazoline) films before and after plasma treatment and additional washing.

#### Fibroblasts adhesion assay

For the cell adhesion study, mice fibroblasts 3T3 were used (DSMZ, Braunschweig, Germany). 3T3 cells were cultivated in Dulbecco’s modified eagle medium (DMEM) supplemented with 10% fetal bovine serum (FBS), streptomycin (100 mg·mL^−1^), penicillin (100 IU·mL^−1^). All chemicals were purchased from Gibco (Life Technologies, Grand Island, NY, USA). The cells were cultivated at 37 °C and 5% CO_2_ with saturating humidity in a CO_2_ incubator. The cells were trypsinized and the medium was changed once every 3 days.

The circular samples in triplicates were sterilized by UV light for 30 min from each side, then transferred to 24 wells plate, covered by 500 μL of media with 20 000 cells per well and incubated for 24 h to assess the cell adhesion. After 24 h, the medium was removed, and the samples were washed by PBS (phosphate-buffered saline). The samples were subsequently stained by fluorescein diacetate (FDA, Invitrogen, 5 μg·mL^−1^ in PBS, stained for 20 min), washed with PBS and visualized using fluorescence microscope (Optika, Italy). From each sample, 5–6 photos from different locations were taken and number of adherent cells was evaluated using an open source imaging processing software (Image J from National Institutes of Health, USA)^65^. The experimental results underwent Kruskall-Wallis non-parametric test followed by post-hoc Dunn’s multiple comparison test to evaluate the differences between tested samples.

## Supplementary information

Supplementary Information.
