# Supplementary material for: Cold atmospheric pressure plasma: simple and efficient strategy for preparation of poly(2-oxazoline)-based coatings designed for biomedical applications
Source: Sci Rep. 2020 Jun 11;10:9478. doi: 10.1038/s41598-020-66423-w (PMC7289869; doi:10.1038/s41598-020-66423-w)
Supplement: Supplementary file 1 — Supplementary Information. [file 41598_2020_66423_MOESM1_ESM.pdf]

Supplementary Information

Cold atmospheric pressure plasma: simple and efficient strategy for preparation of poly(2-oxazoline)-based coatings designed for biomedical applications

Petra Šrámková,[1*] Anna Zahoranová[2], Jakub Kelar[1], Zlata Kelar Tučeková[1], Monika Stupavská[1], Richard Krumpolec[1], Jana Jurmanová[1], Dušan Kováčik[1], Mirko Černák[1]

[1]*Department of Physical Electronics, Faculty of Science, Masaryk University, Kotlarska 2, 611 37 Brno, Czech Republic*

[2]*Polymer Institute, Slovak Academy of Sciences, Dubravska cesta 9, 845 41 Bratislava, Slovakia*

petra.sramkova@mail.muni.cz

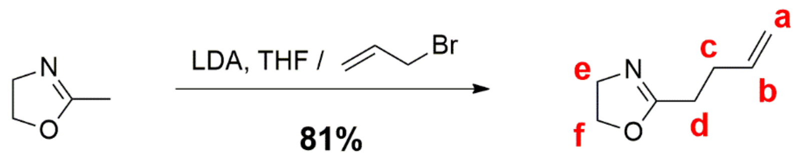

Scheme S1. Synthesis of 2-(3-butenyl)-2-oxazoline (EnOx).

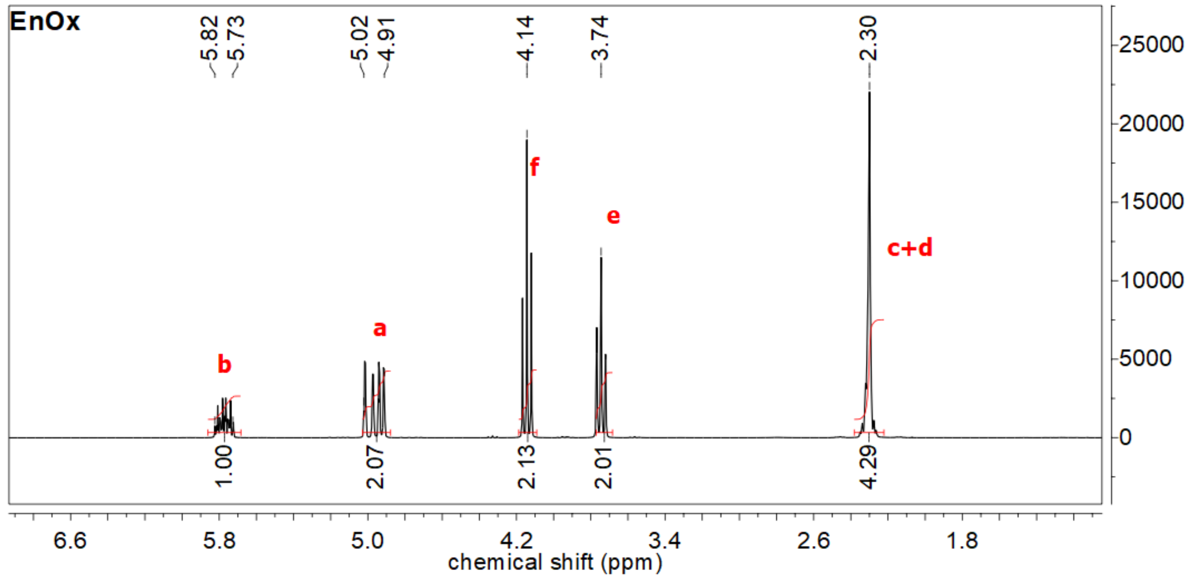

Figure S1. ^{1}H NMR spectrum of 2-(3-butenyl)-2-oxazoline (EnOx).

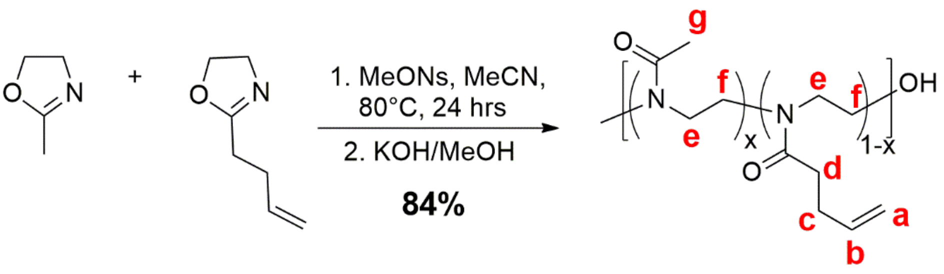

Scheme S2. Synthesis of poly(2-methyl-2-oxazoline)-stat-(2-butenyl-2-oxazoline) (PMEOx).

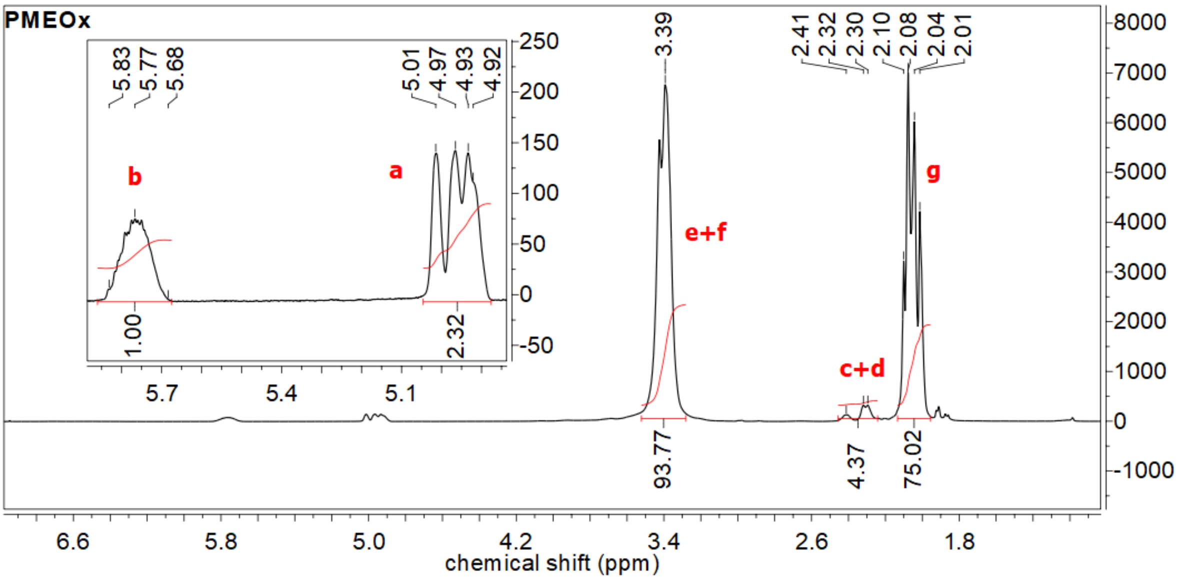

Figure S2. ^{1}H NMR spectrum of poly(2-methyl-2-oxazoline)-stat-(2-butenyl-2-oxazoline) (PMEOx).

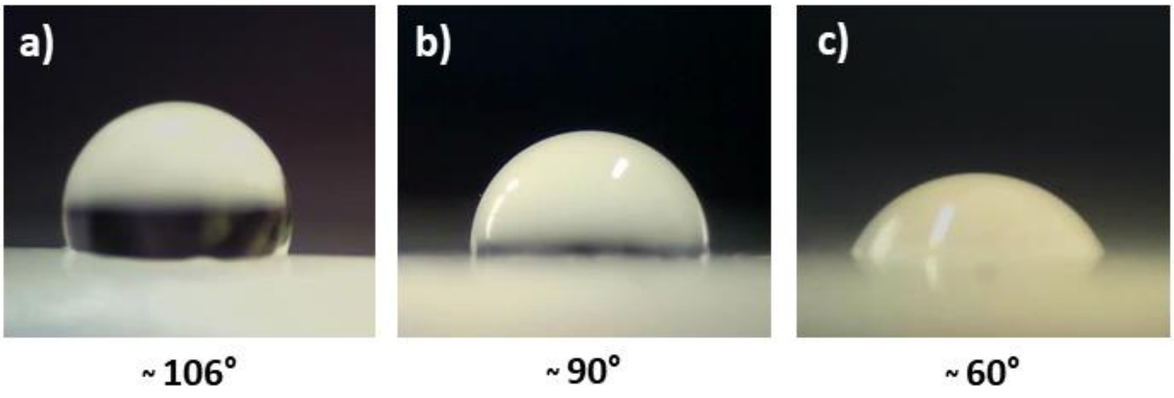

Figure S3. Selected images of water droplets took at the surface of a) raw PTFE, b) air-plasma treated PTFE (3s), and c) PMEOx-coated PTFE after washing (sample PMEOx-air).

Table S1. The atomic concentration and relative percentage of chemical bonds observed from high-resolution C1s spectra of PTFE surface after air-plasma treatment using different plasma exposure times.

	Plasma exposure time [s]					
	REF	3	5	10	30	50
	**atomic concentration [%]**					
**C**	32	33	32	32	33	33
**O**	<1	2	3	8	4	2
**F**	67	65	65	60	63	65
	**C1s bonds concentration [%]**					
**C-C/C-H**	2	10	13	24	4	3
**C-O/C-OH**	1	5	6	9	3	2
**C=O/O-C-O**	<1	2	3	4	4	1
**O-C=O/COOH**	<1	1	1	2	2	1
**CF_X**	96	82	77	61	87	93

Table S2. The atomic concentration and relative percentage of chemical bonds observed from high-resolution C1s spectra of PTFE surface after air-plasma treatment after 10 s after ageing for 28 days.

	Ageing time [days]					
	REF	0	1	7	14	28
	**atomic concentration [%]**					
**C**	32	32	32	32	45	47
**O**	<1	8	8	8	8	8
**F**	67	60	59	59	47	45
	**C1s bonds concentration [%]**					
**C-C/C-H**	2	24	24	24	25	24
**C-O/C-OH**	1	9	10	10	11	12
**C=O/O-C-O**	<1	4	5	6	6	6
**O-C=O/COOH**	<1	2	2	3	3	4
**CF_X**	96	61	59	57	55	54

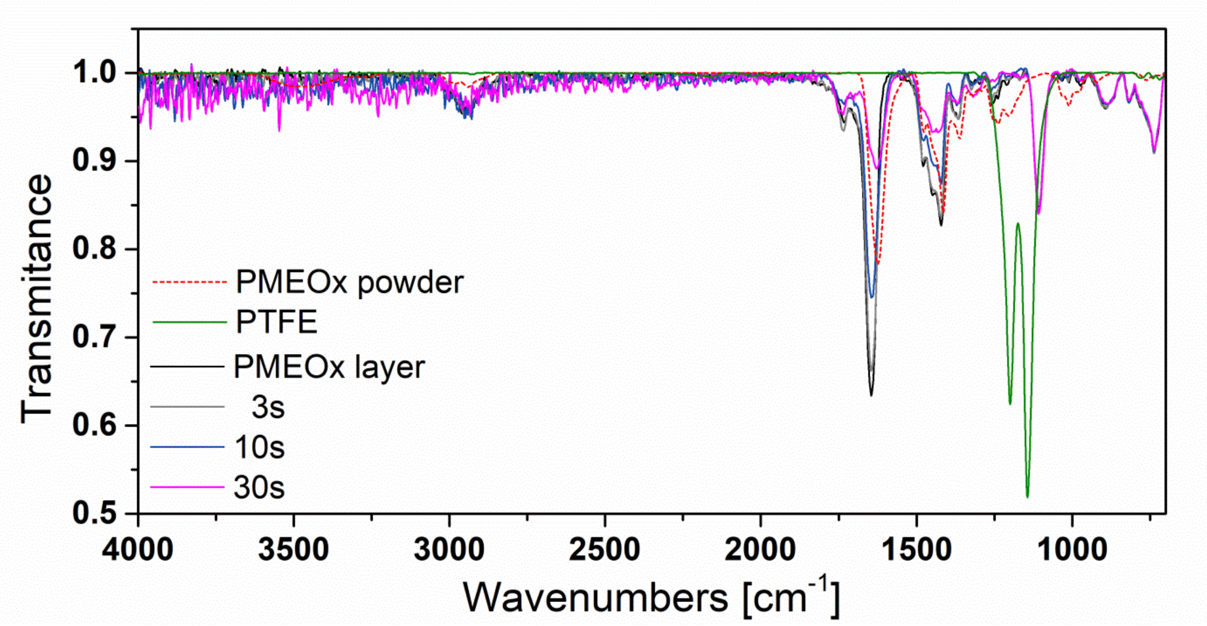

Figure S4. The FTIR spectra of the whole wavelength from 4000 to 600 cm^{-1}. PMEOx powder (red dot line), PTFE substrate (green line), PMEOx layer (black line) and treated PMEOx layer in different air plasma exposure times – 3 s (grey line), 10 s (blue line), 30 s (magenta line), without washing. (***important remark:*** *the spectra of PMEOx layer and plasma-treated PMEOx (3s, 10s, 30s) were measured at Si wafer substrate to avoid the overlapping of signals for C-N (1200-1240 cm^{-1}) with the strong signal for C-C in PTFE spectrum*)

Measurement of PMEOx thickness by SEM

During the preparation of samples, we covered the small part of the sample at the edge by the tape. Then, before the SEM imaging, we removed the tape and compare the samples before and after washing. However, after removing the tape, the edge of the PMEOx layer was irregular and thicker than the rest of the layer as it is depicted at the **Figure S5**. Images a) and d) represent the top view of the sample before (a) and after (d) washing where it is obvious the border of the PMEOx layer and bare PTFE. Images b) and c) are profile pictures, where you can see irregularity and different thicknesses of a layer at the edge, so it was difficult to determine the exact thickness from these SEM images. At picture e) is schematically depicted our visualization of the PMEOx layer after tape removing. Based on the measured thickness at the image c) we conclude that deposited PMEOx layer possessed thickness around 1-3 µm. However, after washing we were not able to measure thickness from the SEM images.

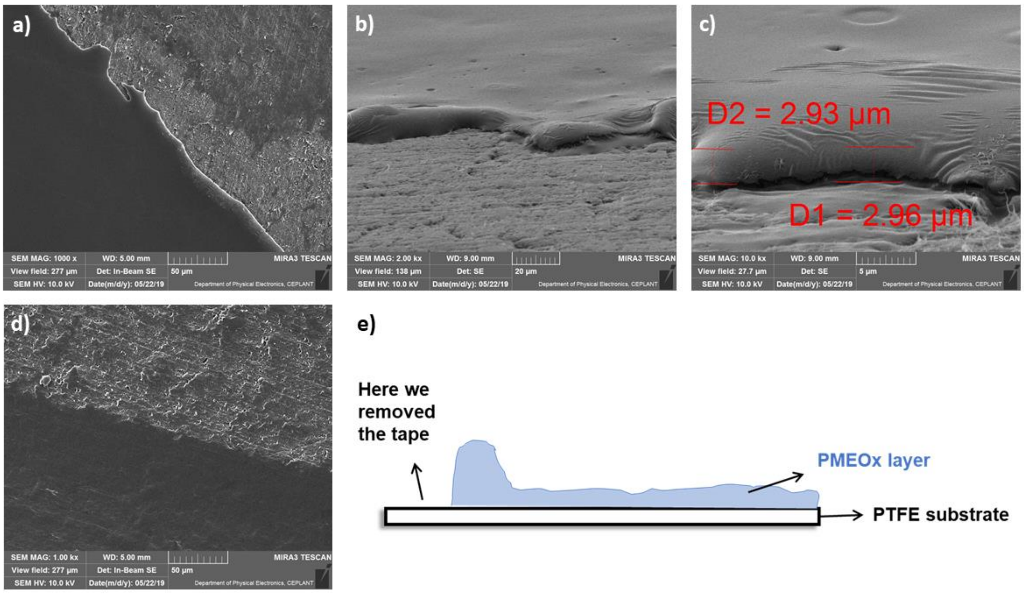

Figure S5 SEM images of a) edge of the PMEOx layer (top view), b) and c) edge of the PMEOx layer (profile view) with estimated thickness, d) edge of the PMEOx layer after washing (top view), e) schematic visualization of the PMEOx layer after tape removing.

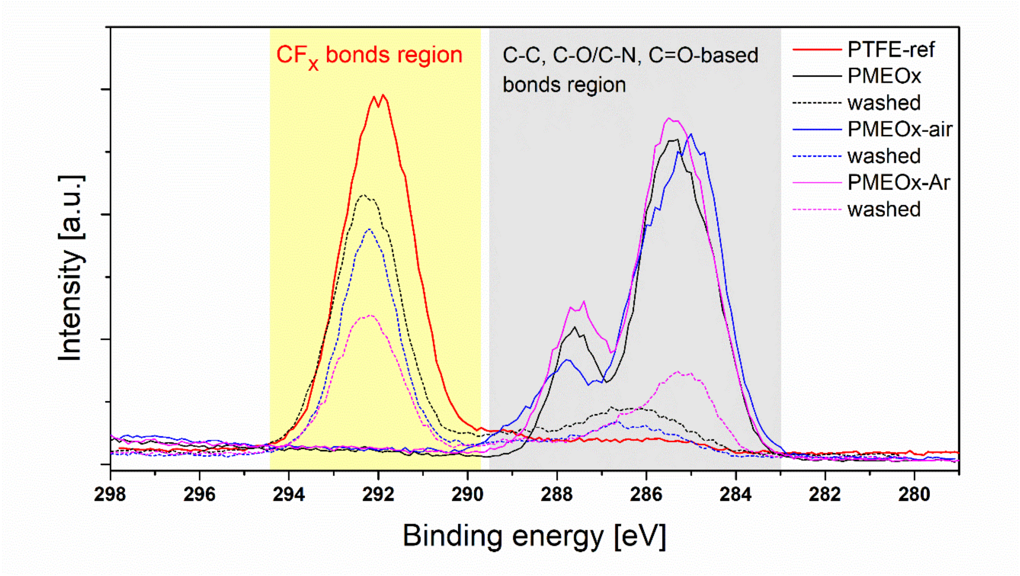

Figure S6. C1s high-resolution spectra of PMEOx coatings before (solid curves) and after washing (dash curves)

Table S3. The chemical composition of washed samples compared to PTFE reference.

	PTEF-ref	PMEOx	PMEOx-air	PMEOx-Ar
	**atomic concentration [%]**			
**C**	32	34	35	40
**O**	<1	4	5	6
**N**	0	2	2	2
**F**	67	60	58	52
**O/C**	0.01	0.12	0.13	0.15
**F/C**	2.1	1.8	1.7	1.3
	**C1s bonds concentration [%]**			
**C-C/C-H**	2	2	2	11
**C-O/C-N**	1	9	10	20
**C=O/N-C=O**	<1	2	3	2
**O-C=O/COOH**	<1	3	2	4
**CF_X**	96	83	82	63
